# Master’s and doctoral degrees in nursing in Brazil: an ecological study

**DOI:** 10.1590/1980-220X-REEUSP-2026-0179en

**Published:** 2026-08-14

**Authors:** Matheus Moraes Silva, José Luís Guedes dos Santos, Gabriela Marcellino de Melo Lanzoni, Alacoque Lorenzini Erdmann

**Affiliations:** 1 Universidade Federal de Santa Catarina, Programa de Pós-Graduação em Enfermagem, Florianópolis, SC, Brazil.

**Keywords:** Nursing, Education, Graduate, Nursing Staff, Teaching, Nursing Research

## Abstract

**Objective:**

To analyze the temporal trend and spatial distribution of master’s and doctoral degrees in the field of nursing in Brazil.

**Method:**

An ecological study using data from the Coordination for the Improvement of Higher Education Personnel, employing descriptive statistics and *Joinpoint* regression for temporal trend analysis.

**Results:**

Between 2004 and 2023, 22,923 degrees were awarded, of which 74.92% were master’s degrees and 25.08% were doctoral degrees, with the highest concentration in the Southeast (49.74%), Northeast (22.20%), and South (20.78%) regions. Master’s degrees showed an upward trend in Brazil (Average Annual Percentage Change—AAPC: 7.55; p < 0.000), with the greatest variation in the North region (AAPC: 16.40; p < 0.000). For doctoral degrees, an upward trend was also observed nationwide (AAPC: 8.31; p < 0.000), particularly in the Midwest (AAPC: 15.96; p < 0.000), while the North region did not record any doctoral degrees at this level during the analyzed period.

**Conclusion:**

The results showed an increasing trend in postgraduate degrees in the field of nursing, coexisting with regional inequalities, which reinforces the need for strategies aimed at expanding and decentralizing postgraduate education to reduce disparities in advanced training.

## INTRODUCTION

Postgraduate Nursing Education (PGENF in the Portuguese acronym) in Brazil was established with the aim of training professionals for teaching in higher education, promoting scientific research, and strengthening evidence-based practices^(1)^. This process was consolidated with the implementation of the first master’s program at the Anna Nery School of Nursing (EEAN) in 1972 and the doctoral program at the University of São Paulo School of Nursing (EEUSP) in 1981^(1,2)^.

In recent decades, graduate education in the field has grown exponentially. Data from the Coordination for the Improvement of Higher Education Personnel (CAPES) indicate that, as of 2024, the country had 86 programs, totaling 135 courses, with 58 programs and 99 courses in the academic track and 28 programs and 36 courses in the professional track. The academic track prioritizes the production of scientific knowledge, training for research, and qualification for teaching, while the professional track emphasizes the application of knowledge in practice and the development of technologies and innovations in healt^(3)^.

Graduate Nursing Programs (PPGENFs in the Portuguese acronym) are primarily responsible for producing specialized knowledge in the field and contribute substantially to the advancement of nursing science^(4)^, a position reflected in Brazil’s first-place ranking in nursing scientific output in Latin America and among the top ten countries globally, according to the 2024 SCImago *ranking* ^(5)^.

Against this background, it is important to understand how master’s and doctoral degrees in nursing have been configured from temporal and spatial perspectives within Brazil. Although the expansion of Brazilian PPGENFs is widely recognized, studies that take the degrees awarded as their central object of analysis remain scarce, especially considering their regional distribution and evolution over time. In this context, investigating this scenario can contribute to identifying disparities in advanced nursing education, informing reflections related to the planning of PPGENFs, human resource development, and scientific and educational policies in the field.

Given the above, this study aims to analyze the temporal trend and spatial distribution of master’s and doctoral degrees in the field of nursing in Brazil.

## METHOD

### Study Design and Location

This is an ecological study based on secondary data extracted from CAPES. In this type of design, inferences are restricted to the population level^(6)^. The units of analysis were the 27 states and the five geographic regions of Brazil. The study was reported in accordance with the STROBE (*Strengthening the Reporting of Observational Studies in Epidemiology*) guidelines.

### Sample, Selection Criteria, and Data Source

The study included all records of master’s and doctoral degrees in nursing available in the CAPES Open Data Panel^(7)^. Data collection was performed on November 15, 2025, by selecting the topic “Evaluation of *Stricto Sensu* Graduate Programs” and the group “*Stricto Sensu* Graduate Students in Brazil.” Files in CSV (*Comma-Separated Values*) format were extracted for the period from 2004 to 2023, corresponding to the complete time interval available in the database at the time of collection. For data processing, the “field of study” variable was initially filtered, selecting only records classified as “Nursing” and excluding all others. Next, the data extracted from the different spreadsheets were aggregated into a single database for organization and analysis. After aggregation, the records were reviewed for consistency of the analyzed variables, and no missing data were identified.

### Study Variables

The extracted variables were: year of degree conferral; age group of the graduate; country of nationality; academic degree (academic master’s, professional master’s, academic doctorate, or professional doctorate); and granting institution of higher education (IHE). In the trend analysis, the dependent variable was the annual percentage of degrees awarded, calculated as the ratio of the number of graduates in each year to the total number of degrees awarded at the respective educational level during the analyzed period, multiplied by 100. The independent variable was the year of graduation.

### Data Analysis and Processing

The data were organized in *Microsoft Excel* ^®^. Descriptive statistics were calculated using the JASP *software* (version 0.95.4; Amsterdam, Netherlands). The spatial distribution of degrees was represented using choropleth maps created in QGIS software (version 3.44.4), utilizing *shapefiles* of the federal units provided by the Brazilian Institute of Geography and Statistics (IBGE). The data were aggregated by state and represented using absolute and relative frequencies, allowing for the visualization of the spatial distribution of degrees across Brazil. Additionally, in each region, the two HEIs that awarded the most master’s and doctoral degrees in the field of nursing were highlighted.

The temporal trend analysis was conducted using independent annual series for master’s and doctoral degrees in the *Joinpoint Regression Program* (version 5.3.0). The method employs segmented regression with logarithmic transformation of the values and the Monte Carlo permutation test, with a significance level of p < 0.05, to identify points of change over the analyzed period^(8)^. Trends were expressed as the Average Annual Percentage Change (AAPC), with a 95% confidence interval (95% CI). The AAPC is a summary measure of the trend over a predefined time interval, calculated as the weighted average of the annual percentage changes from the inflection point model^(9)^.

The AAPC formula is expressed as: AAPC = {exp (Σw_i_/Σwi) − 1} × 100, where w_i_ represents the regression coefficient for each time interval identified by the model and w_i_, the width of the interval, defined by the number of years in each segment^(9)^. Negative AAPC values indicate a decreasing trend, and positive values indicate an increasing trend, provided they are statistically significant (p < 0.05). In the absence of statistical significance (p > 0.05), the series is interpreted as stationary^(10)^.

### Ethical Considerations

As this is a study conducted using secondary data in the public domain with unrestricted access, approval by a Research Ethics Committee was not required, as established in Resolution No. 510/2016 of the National Health Council^(11)^.

## RESULTS

Between 2004 and 2023, 22,923 postgraduate degrees in the field of nursing were identified in Brazil, of which 17,174 (74.92%) were master’s degrees and 5,749 (25.08%) were doctoral degrees, with the highest concentration in the Southeast (49.74%), Northeast (22.20%), and South (20.78%) regions. Among the degree holders, the age groups of 30 to 39 years (n = 10,390; 45.32%) and 20 to 29 years (n = 6,789; 29.62%) predominated (Table 1).

**Figure 1:**
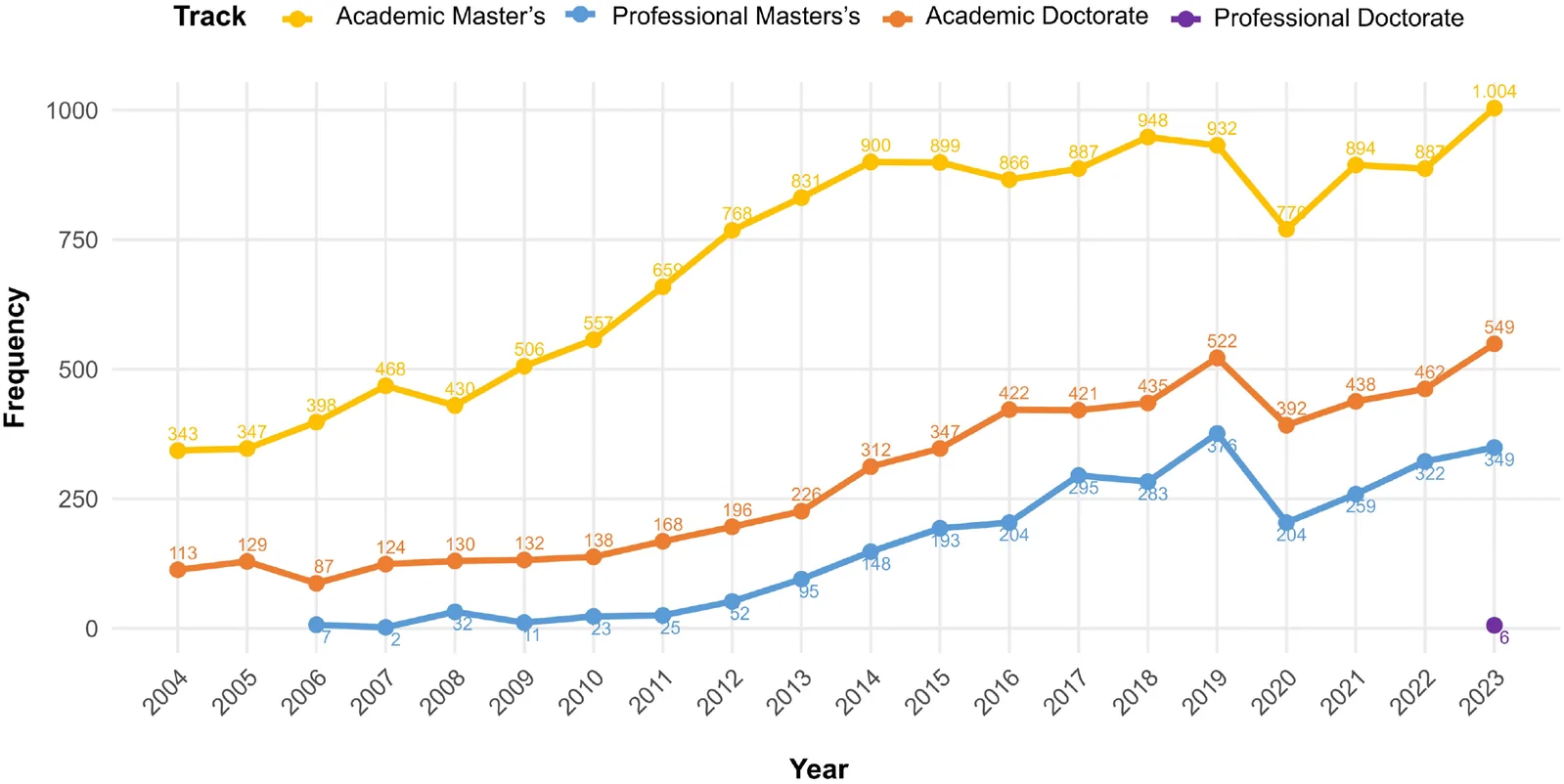
Nursing graduates, by academic degree (n = 22,923) – Brazil, 2004–2023.

Regarding academic degrees, higher proportions were observed for academic master’s degrees (n = 14,294; 62.36%) and academic doctoral degrees (n = 5,743; 25.05%). Regarding the legal status of the granting institutions, the majority of degrees were awarded by federal public institutions (n = 13,654; 59.56%) (Table 1). Figure 1 presents the temporal distribution of degrees by type during the analyzed period, highlighting the annual variation in the number of degrees awarded throughout the historical series, with a reduction observed in 2020.

**Table 1:** Distribution of degree holders by region, age group, academic degree, and institutional status – Brazil, 2004–2023.

Variables	Brazilian Region					Brazil
	North	Northeast	Midwest	Southeast	South	
	n = 362 (%)	n = 5,090 (%)	n = 1,304 (%)	n = 11,403 (%)	n = 4,764 (%)	n = 22,923 (%)
*Age group of graduates*						
19 or younger	0 (0.00)	0 (0.00)	0 (0.00)	0 (0.00)	1 (0.03)	1 (0.00)
20 to 29 years	134 (37.02)	1,776 (34.88)	477 (36.58)	2,836 (24.88)	1,468 (30.82)	6,789 (29.62)
30 to 39 years	156 (43.10)	2,230 (43.79)	572 (43.86)	5,303 (46.51)	2,129 (44.68)	10,390 (45.32)
40 to 49 years	52 (14.36)	747 (16.67)	211 (16.19)	2,338 (20.50)	860 (18.05)	4,208 (18.36)
50 to 59 years	17 (4.70)	199 (3.90)	38 (2.91)	793 (6.96)	275 (5.77)	1,322 (5.77)
60 to 69 years	3 (0.82)	35 (0.68)	6 (0.46)	125 (1.09)	30 (0.62)	199 (0.87)
70 or more	0 (0.00)	5 (0.08)	0 (0.00)	8 (0.06)	1 (0.03)	14 (0.06)
*Academic degree*						
Academic master’s	306 (84.53)	3,377 (66.35)	987 (75.69)	6,852 (60.09)	2,772 (58.19)	14,294 (62.36)
Professional Master’s	56 (15.47)	676 (13.28)	148 (11.35)	1,134 (9.94)	866 (18.18)	2,880 (12.56)
Academic doctorate	0 (0.00)	1,037 (20.37)	169 (12.96)	3,414 (29.94)	1,123 (23.57)	5,743 (25.05)
Professional doctorate	0 (0.00)	0 (0.00)	0 (0.00)	3 (0.03)	3 (0.06)	6 (0.03)
*Classification of the legal status of the institution offering the graduate program*						
Federal	225 (62.15)	3,559 (69.92)	1,016 (77.91)	5,274 (50.27)	3,580 (75.15)	13,654 (59.56)
State	137 (37.85)	1,172 (23.03)	148 (11.35)	5,732 (46.25)	885 (18.58)	8,074 (35.23)
Private	0 (0.00)	359 (7.05)	140 (10.74)	397 (3.48)	299 (6.28)	1,195 (5.21)

**Figure 2:**
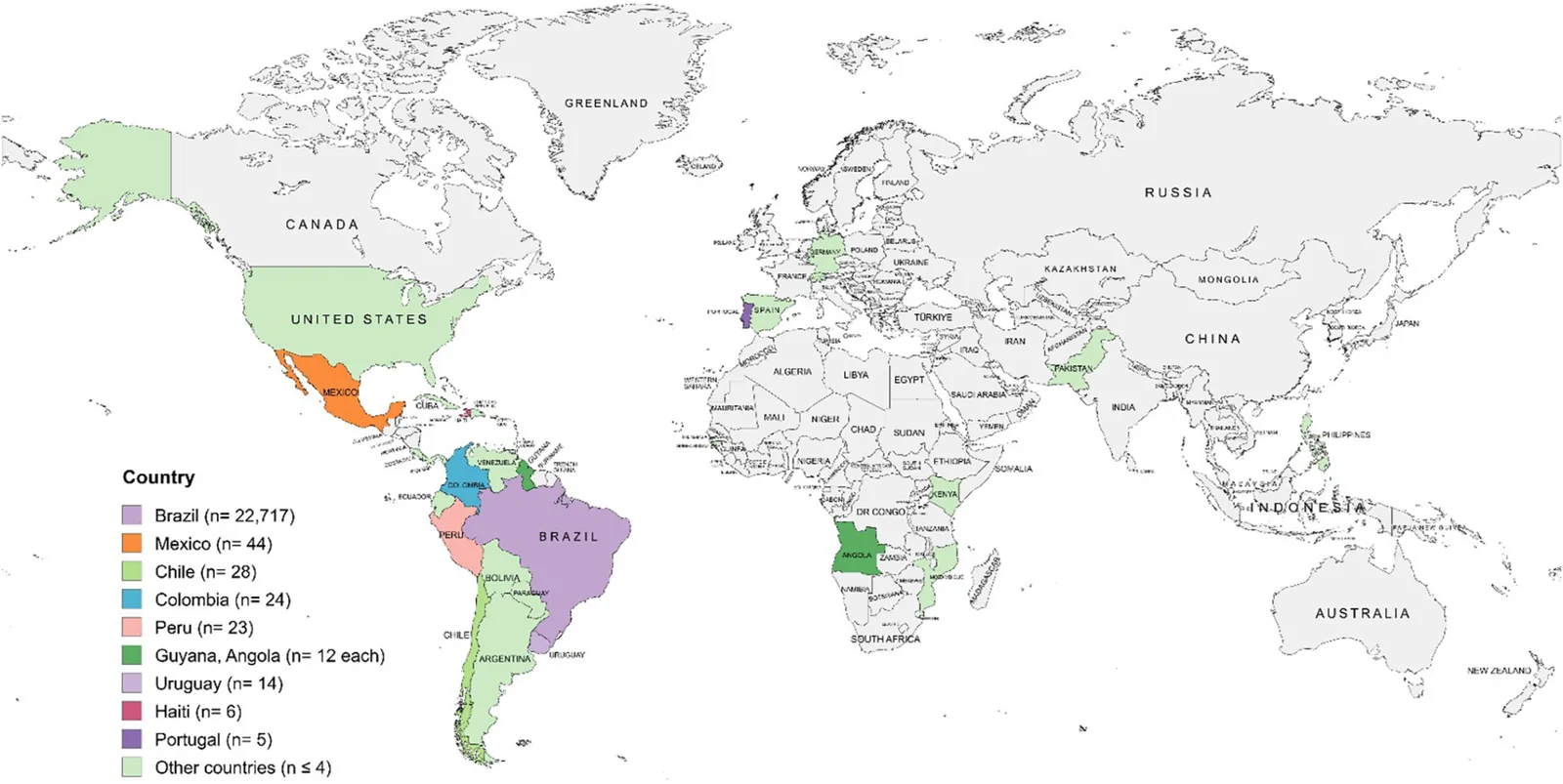
Distribution of nursing degree holders in Brazil by country of nationality, 2004–2023.

Among the higher education institutions that awarded the most master’s and doctoral degrees, the Federal University of Pará (n = 133) and the State University of Pará (n = 102) stood out in the North region. In the Northeast, the highest numbers were observed at the Federal University of Ceará (n = 1,130) and the Federal University of Paraíba (n = 654). In the Midwest, the Federal University of Goiás (n = 418) and the Federal University of Mato Grosso (n = 226) stood out. In the Southeast, the programs were concentrated at the University of São Paulo (n = 4,012) and the Federal University of Rio de Janeiro (n = 903). In the South, the Federal University of Santa Catarina (UFSC) (n = 1,118) and the Federal University of Paraná (n = 571) stood out. Regarding the nationality of the graduates, 92.86% (n = 22,717) were Brazilian and 7.14% (n = 206) were foreign nationals from 29 countries. Among the foreign nationals, 98 earned a master’s degree and 108 a doctoral degree. The highest numbers were observed among graduates from Mexico (n = 44), Chile (n = 28), Colombia (n = 24), and Peru (n = 23) (Figure 2).

**Figure 3:**
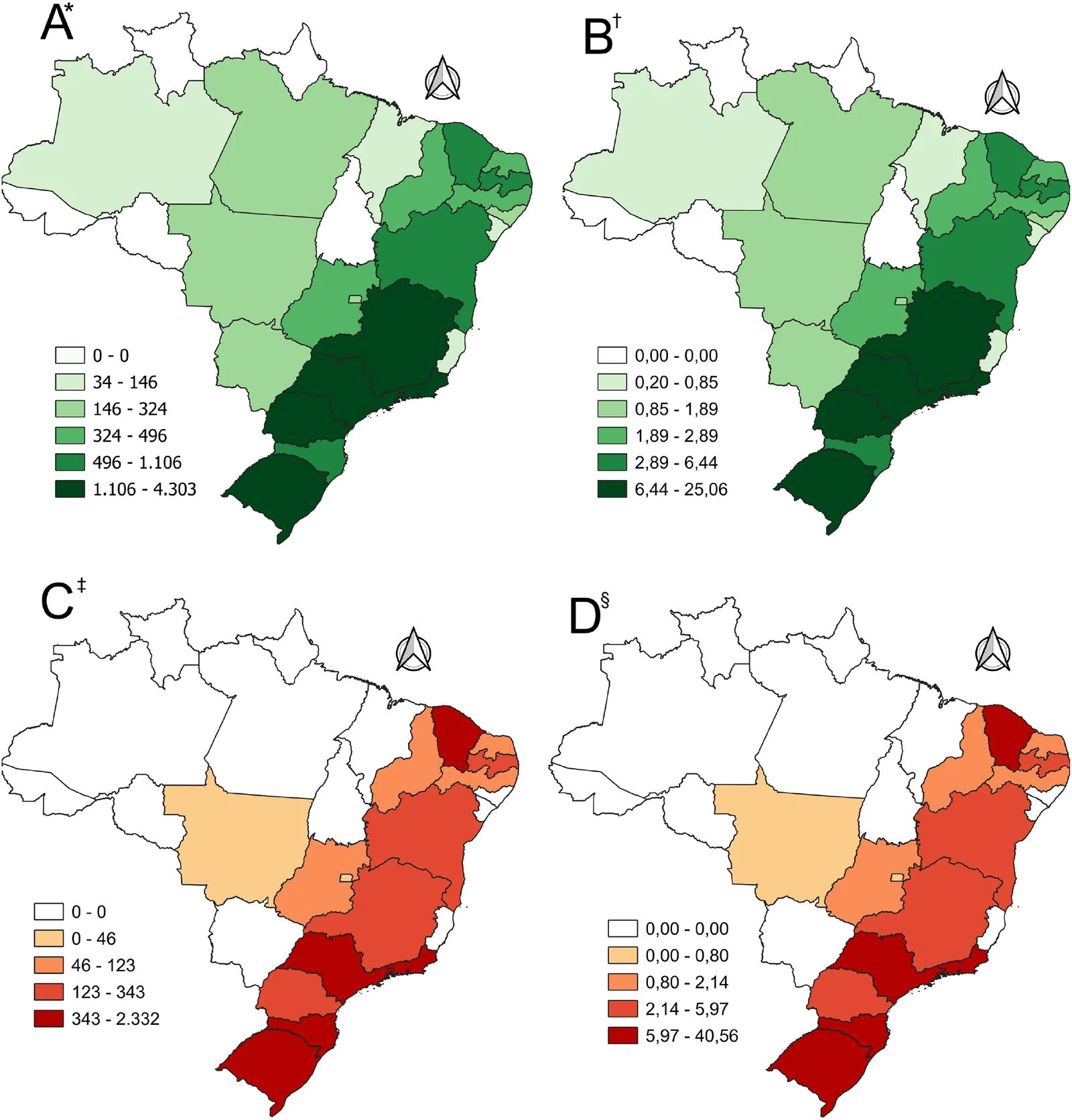
Spatial distribution of master’s and doctoral degrees in the field of nursing by state – Brazil, 2004–2023.

The spatial distribution of master’s degrees showed a marked concentration in the South and Southeast regions, especially in the states of São Paulo, Rio de Janeiro, Minas Gerais, Rio Grande do Sul, and Paraná, which were in the highest intensity category. In the Northeast, Bahia, Ceará, and Paraíba stood out. In contrast, Espírito Santo, Sergipe, Amazonas, Maranhão, and most states in the North region had low numbers, while Acre, Amapá, Rondônia, Roraima, and Tocantins did not record any master’s degrees during the period analyzed (Figures 3A and 3B)

*A = absolute frequency (master’s degree); ^†^B = relative frequency (master’s degree); ^‡^C = absolute frequency (doctoral degree); ^§^D = relative frequency (doctoral degree)

With regard to doctoral degrees, Figures 3C and 3D show a concentration in the states of São Paulo, Rio de Janeiro, Rio Grande do Sul, and Santa Catarina. In the Northeast, Ceará stood out as the main hub for training, while Bahia and Paraíba showed intermediate levels. In contrast, no doctoral degrees in nursing awarded by local institutions were recorded in the states of the North region (Acre, Amapá, Amazonas, Pará, Rondônia, Roraima, and Tocantins) nor in the states of Maranhão, Alagoas, Sergipe, Espírito Santo, and Mato Grosso do Sul during the analyzed period (Figures 3C and 3D).

Over the 20-year period analyzed, master’s degrees in nursing showed a statistically significant upward trend (AAPC: 7.55%; p < 0.000), with growth recorded in all five regions, particularly in the North region (AAPC: 16.40%; p < 0.000). For doctoral degrees, an equally increasing and significant national trend was observed (AAPC: 8.31% per year; p < 0.000), noted in four regions, with the highest AAPC in the Midwest (15.96%; p < 0.000), as shown in Table 2.

**Table 2:** Average annual percentage change in master’s and doctoral degrees in the field of nursing in Brazil and by region – Brazil, 2004–2023.

Variables	Period	AAPC*	Trend	95% CI^†^	^‡^ p-value
*Master’s*					
Brazil	2004–2023	7.55	Upward	6.60; 8.90	< 0.000
North	2012–2023	16.40	Upward	9.82; 26.57	< 0.000
Northeast	2004–2023	10.78	Upward	9.54; 12.55	< 0.000
Midwest	2004–2023	14.23	Upward	12.70; 17.19	< 0.000
Southeast	2004–2023	4.48	Upward	3.56; 5.53	< 0.000
South	2004–2023	9.25	Upward	6.86; 13.67	< 0.000
*Doctorate* ^§^					
Brazil	2004–2023	8.31	Upward	6.89; 9.72	< 0.000
Northeast	2004–2023	15.53	Upward	13.14; 20.49	< 0.000
Midwest	2013–2023	15.96	Upward	8.21; 29.73	< 0.000
Southeast	2004–2023	6.44	Upward	4.31; 9.16	< 0.000
South	2004–2023	14.67	Upward	12.13; 20.80	< 0.000

*AAPC = Annual Average Percentage Change;

^†^ 95% CI = 95% Confidence Interval;

^‡^ p < 0.05;

^§^ PhD = The North region was not included due to the absence of PhDs in the field of nursing during the period under analysis.

## DISCUSSION

The data revealed a high concentration of master’s and doctoral degrees in the Southeast, Northeast, and South regions, with a predominance of the academic track and greater participation by federal public higher education institutions in awarding these degrees. This pattern is consistent with the nursing field document, which points to the centralization of PPGENFs in these regions and the prevalence of the academic track^(3)^. Federal public HEIs account for the largest share of stricto sensu programs in the country, which helps explain their central role in training master’s and doctoral students in this study^(12)^.

The leading role of the Southeast region in stricto sensu nursing education can be understood through its historical process of institutionalization, considering that the first PPGENFs were established in this region and subsequently expanded to the South and Northeast regions^(1)^. From this perspective, the distribution of degrees observed in the results reflects historical trajectories of consolidation and expansion of graduate education in the field, since regions with earlier institutionalization tend to concentrate a greater volume of programs and, consequently, of degrees. This trajectory is part of a broader process of expansion of PGENF in the Americas, whose first programs were initiated in the United States (U.S.) in the 1930s, later expanding to Latin American countries. In this regard, the American influence on the consolidation of Brazilian PGENF stands out, evidenced by the specialization of Brazilian faculty in the US and by the financial support of institutions such as the Kellogg Foundation for both human resource training and the structuring of the first programs in the country^(2)^.

In the North region, the first master’s program in nursing began only in 2010, through a partnership between the State University of Pará (UEPA) and the Federal University of Amazonas (UFAM)^(13)^, highlighting a historical disparity spanning several decades compared to the Southeast, South, and Northeast regions, where master’s programs had already been in operation since the 1970s^(1)^. Furthermore, during the period analyzed, no doctoral degrees in nursing were awarded by institutions in the region, since the first program at this level was approved only in 2023, also linked to UEPA/UFAM, with the first cohort beginning in 2024. The implementation of this program constitutes a milestone in the consolidation of advanced regional education, by expanding the qualification of human resources at the doctoral level and strengthening scientific production and innovation in nursing in the Amazonian context^(14,15)^. The nascent nature of Professional Doctorate (DP) degrees observed in this study can be explained by the recent implementation of this modality in Brazilian nursing. DP programs began in 2019, while the first defenses took place only in 2023, at UFSC and the São Paulo State University (UNESP)^(16)^.

The regional disparities identified are not new and are recognized by the field itself, which points to the persistence of challenges related to the concentration of programs in state capitals and the low availability of courses in the North, the interior of the Northeast, and the Midwest. Strategies to reduce these disparities include expanding graduate programs to the interior, increasing the availability of doctoral programs, and strengthening inductive strategies, such as the Interinstitutional Master’s and Doctoral Programs (called Minter and Dinter respectively) and establishing partnerships between PPGENFs at different levels of maturity^(3)^.

In this study, a reduction in the number of degrees awarded was observed in 2020. This finding is not an isolated phenomenon, given that evidence from Brazilian graduate education points to a decrease in degrees awarded across different fields, associated with difficulties in completing programs during the COVID-19 pandemic^(17)^. Nursing data from the study *Brasil: mestres e doutores* (*Brazil: Masters and Doctoral degree holders)* corroborate this picture, indicating a reduction in the growth rates of degrees awarded in 2020, both for master’s degrees (–25.54%) and doctoral degrees (–24.9%)^(18)^. In light of this context, the observed decrease may have been influenced by institutional measures adopted by CAPES, such as the relaxation of degree completion deadlines and the exceptional extension of scholarships during the pandemic^(19, 20, 21)^. At the same time, the psychosocial impacts of the pandemic also proved significant, associated with effects on the mental health of Brazilian graduate students, such as demotivation, difficulty concentrating, insomnia, and high levels of anxiety and depression, as well as setbacks in academic production and meeting deadlines—factors that may have influenced academic progression^(22, 23, 24)^.

Regarding the nationality of degree recipients, the data indicate that Brazil has contributed significantly to the training of foreign master’s and doctoral students, especially those from Latin America. The development of inter-institutional graduate programs constitutes one of the main ways in which this contribution can occur^(25)^. In this context, the nursing field reaffirms its commitment to continue contributing in solidarity with proposals from the Pan American Health Organization (PAHO) and the World Health Organization (WHO) aimed at training PhD nurses for Latin America and the Caribbean, including the adoption of mechanisms to attract students from the region and the strengthening of South–South integration, with a view to establishing a core of PhDs in their countries of origin and promoting scientific and technological development in the region^(3)^.

In the Ibero-American context, doctoral education in nursing has been characterized by the progressive expansion of programs, accompanied by persistent regional inequalities in their availability. In this scenario, Brazil stands out as a pioneer in this educational modality, having begun in 1982, and as the main regional hub, concentrating the largest number of active PPGENFs^(26)^. Specifically within Latin America, there has been growth in the number of programs, with Brazil, Peru, and Mexico accounting for the majority of the programs offered. Despite this expansion, challenges persist that compromise the quality and sustainability of doctoral education, namely: the unequal geographic distribution of programs, limited research funding, low inter-institutional coordination, and the prominence of doctoral programs primarily oriented toward academic research, with limited inclusion of models focused on advanced practice^(27)^.

From this perspective, the concentration of PhDs in Latin America may represent a relevant strategy for reducing disparities in advanced nursing education, especially in countries with a still-limited supply of doctoral programs. The training of these professionals in Brazilian programs may foster the expansion of regional capacity for research and stricto sensu education. However, the impacts of this process depend on structural conditions in the countries of origin, including policies for professional recognition, scientific funding, and institutional support for the consolidation of new graduate programs.

Beyond these aspects, the literature highlights the need to intensify international cooperation and the development of multicenter research as strategies for strengthening doctoral education in nursing and for consolidating the field in different contexts^(28)^. In this same vein, inequalities are evident that extend beyond the territorial dimension, reaching social and racial aspects, with persistent barriers for minority populations and gaps in support for students with intersectional identities, which reinforces the centrality of equity as a structural pillar of doctoral education in nursing^(29)^.

Master’s and doctoral-level education in nursing must be understood as extending beyond the mere attainment of a degree, involving the development of individuals capable of exercising scientific, political, and social leadership, producing relevant knowledge, and responding to the contemporary challenges of the profession and society. In this context, graduate nursing education is situated within a landscape of profound scientific, technological, and social transformations, demanding master’s and doctoral graduates committed to innovation, sustainability, equity, and the generation of concrete impacts in care, management, and health policy formulation. Thus, the expansion and consolidation of stricto sensu education must be accompanied by continuous reflection on the role of these professionals in building the science of nursing and in strengthening their ethical, social, and political commitment^(30)^.

This study demonstrated a significant increase in master’s and doctoral degrees in the country over the analyzed period, including in regions historically underserved by program offerings. This progress, though significant, raises questions that go beyond the quantitative dimension of advanced education. How many graduates would be needed to promote a more equitable distribution of education across the country? And, above all, would increasing the number of master’s and doctoral graduates be sufficient to address the regional disparities historically entrenched in Brazilian nursing?

These questions highlight contradictions, uncertainties, and challenges inherent in the historical evolution of the nursing profession in Brazil, the trajectory of university education, and the paths of consolidation of science and technology in nursing. Similarly, they reveal tensions regarding the very model of postgraduate expansion, since the impact of degree conferral does not depend exclusively on the volume of trained professionals, but on broader structural conditions, such as policies for retaining and valuing master’s and doctoral graduates, sustainable research funding, opportunities for qualified entry into teaching, practice, and health management, as well as the capacity of regions to absorb and value these professionals. In this sense, stricto sensu training, although fundamental, does not automatically guarantee the reduction of regional inequalities nor the production of proportional impacts in professional practice, requiring coordination among educational, scientific, health, and regional development policies. Thus, the challenge is not merely to train more professionals, but to create conditions for master’s and doctoral graduates to produce concrete results in these different fields, with a view to achieving high scientific and technological mastery and consolidating Brazilian nursing as a benchmark on the international stage.

This study has some limitations. The first relates to the use of secondary data, which may entail restrictions regarding the quality of the available information. Furthermore, the use of this type of database may limit more in-depth analyses due to the restriction of available variables. Furthermore, trend analysis based on degree completion rates may limit the understanding of the magnitude of variations observed over time. Another limitation concerns the analysis of aggregated data by state and region, which may obscure the identification of inequalities at smaller geographic scales, such as municipalities. Given this, future studies may employ different methodological designs, additional variables, and complementary analyses using absolute values or adjusted rates to broaden the understanding of the phenomenon.

The time frame covered by the database was limited to the period from 2004 to 2023, not encompassing the entirety of graduate-level nursing education in the Brazilian context. Added to this is the scarcity of studies on the temporal evolution of nursing degrees, especially from an international perspective, which limited more in-depth comparisons between the findings of this study and contexts with a well-established tradition in postgraduate nursing education. Nevertheless, the analyzed data allowed for a consistent assessment of the evolution of nursing degrees over two decades, offering relevant insights for understanding the dynamics of advanced education in the field.

## CONCLUSION

This study analyzed the temporal trend and spatial distribution of master’s and doctoral degrees in the field of nursing, demonstrating growth over the period in all regions of Brazil. The findings indicate a predominance of master’s-level degrees, mostly in the academic modality, and the central role of federal public higher education institutions in awarding these degrees. From a spatial perspective, a concentration of education was observed in the Southeast, Northeast, and South regions, as well as the absence of master’s and doctoral degrees awarded by local institutions in some states over the analyzed period, especially in the North region.

Despite the observed growth, marked regional disparities persist in stricto sensu nursing education. This scenario reinforces the need for strategies aimed at decentralizing PPGENFs, expanding course offerings in historically underserved regions, and bringing advanced education to rural areas. The data presented can, in this sense, assist academic administrators and educational policymakers in defining actions aimed at strengthening programs in the process of consolidation and at introducing new training offerings at the master’s and doctoral levels. Furthermore, the expansion of degree programs must be accompanied by policies to value, retain, and effectively integrate master’s and doctoral graduates into local communities, thereby maximizing impacts on teaching, research, professional practice, and health management.

## Data Availability

The entire dataset supporting the results of this study is available upon request to the corresponding author.
