# Supplementary material for: Master’s and doctoral degrees in nursing in Brazil: an ecological study
Source: Rev Esc Enferm USP. 2026 Aug 14;60:e20260179. doi: 10.1590/1980-220X-REEUSP-2026-0179en (PMC13489687; doi:10.1590/1980-220X-REEUSP-2026-0179en)
Supplement: Translation

# Translation: Titulações de mestrado e doutorado na área de enfermagem no Brasil: estudo ecológico

**DOI:** 10.1590/1980-220X-REEUSP-2026-0179pt

**Authors:** Matheus Moraes Silva, José Luís Guedes dos Santos, Gabriela Marcellino de Melo Lanzoni, Alacoque Lorenzini Erdmann

**Affiliations:** 1 Universidade Federal de Santa Catarina, Programa de Pós-Graduação em Enfermagem, Florianópolis, SC, Brasil.

**Keywords:** Enfermagem, Educação de Pós-Graduação, Recursos Humanos de Enfermagem, Ensino, Pesquisa em Enfermagem

## Abstract

**Objetivo:**

Analisar a tendência temporal e a distribuição espacial das titulações de mestrado e doutorado na área de enfermagem no Brasil.

**Método:**

Estudo ecológico com dados da Coordenação de Aperfeiçoamento de Pessoal de Nível Superior, empregando estatística descritiva e regressão *Joinpoint* para análise de tendência temporal.

**Resultados:**

Entre 2004 e 2023, foram registradas 22.923 titulações, sendo 74,92% de mestrado e 25,08% de doutorado, com maior concentração nas regiões Sudeste (49,74%), Nordeste (22,20%) e Sul (20,78%). As titulações de mestrado apresentaram tendência crescente no Brasil (Variação Percentual Anual Média-AAPC: 7,55; p < 0,000), com maior variação na região Norte (AAPC: 16,40; p < 0,000). Para o doutorado, verificou-se igualmente tendência crescente no país (AAPC: 8,31; p < 0,000), com destaque para o Centro-Oeste (AAPC:15,96; p < 0,000), enquanto a região Norte não registrou titulações nesse nível no período analisado.

**Conclusão:**

Os resultados evidenciaram tendência crescente das titulações de pós-graduação na área de enfermagem, coexistindo com desigualdades regionais, o que reforça a necessidade de estratégias voltadas à ampliação e à descentralização da pós-graduação para redução das assimetrias na formação avançada.

## INTRODUÇÃO

A Pós-Graduação em Enfermagem (PGENF) no Brasil foi instituída com o objetivo de qualificar profissionais para a docência no ensino superior, impulsionar a produção científica e fortalecer as práticas baseadas em evidências^(1)^. Esse processo consolidou-se com a implantação do primeiro mestrado na Escola de Enfermagem Anna Nery (EEAN), em 1972, e do doutorado na Escola de Enfermagem da Universidade de São Paulo (EEUSP), em 1981^(1,2)^.

Nas últimas décadas, a pós-graduação na área cresceu exponencialmente. Dados da Coordenação de Aperfeiçoamento de Pessoal de Nível Superior (CAPES) indicam que, até 2024, o país contava com 86 programas, totalizando 135 cursos, sendo 58 programas e 99 cursos na modalidade acadêmica e 28 programas e 36 cursos na modalidade profissional. A modalidade acadêmica prioriza a produção de conhecimento científico, a formação para a pesquisa e a qualificação para a docência, enquanto a profissional enfatiza a aplicação do conhecimento na prática e o desenvolvimento de tecnologias e inovações nos serviços de saúde^(3)^.

Os Programas de Pós-Graduação em Enfermagem (PPGENFs) são os principais responsáveis pela produção de conhecimento especializado na área e contribuem substancialmente para o avanço da ciência da enfermagem^(4)^, posição que se reflete no primeiro lugar do Brasil na produção científica em enfermagem na América Latina e entre os dez países de maior produção mundial, conforme o *ranking* SCImago de 2024^(5)^.

À luz desse cenário, torna-se relevante compreender como as titulações de mestrado e doutorado em enfermagem têm se configurado sob as perspectivas temporal e espacial no território brasileiro. Embora a expansão da PGENF brasileira seja amplamente reconhecida, ainda são escassos os estudos que adotam as titulações concedidas como objeto central de análise, especialmente considerando sua distribuição regional e evolução ao longo do tempo. Nesse contexto, investigar esse cenário pode contribuir para identificação de assimetrias na formação avançada em enfermagem, subsidiando reflexões relacionadas ao planejamento dos PPGENFs, à formação de recursos humanos e às políticas científicas e educacionais da área.

Diante do exposto, este estudo tem como objetivo analisar a tendência temporal e a distribuição espacial das titulações de mestrado e doutorado na área de enfermagem no Brasil.

## MÉTODO

### Desenho e Local de Estudo

Trata-se de um estudo ecológico desenvolvido com base em dados secundários extraídos da CAPES. Nesse tipo de delineamento, as inferências restringem-se ao nível populacional^(6)^. As unidades de análise corresponderam aos 27 estados e às cinco regiões geográficas do Brasil. O estudo foi reportado de acordo com as recomendações do STROBE (*Strengthening the Reporting of Observational Studies in Epidemiology*).

### Amostra, Critérios de Seleção e Fonte de Dados

O estudo foi constituído por todos os registros de titulação de mestres e doutores em enfermagem disponíveis no Painel de Dados Abertos da CAPES^(7)^. A coleta de dados foi realizada em 15 de novembro de 2025, mediante seleção do tema “Avaliação da Pós-graduação *Stricto Sensu*” e do grupo “Discentes da Pós-graduação *Stricto Sensu* no Brasil”. Foram extraídos arquivos no formato CSV (*Comma-Separated Values*) referentes ao período de 2004 a 2023, correspondente ao intervalo temporal completo disponível na base no momento da coleta. Para o tratamento dos dados, procedeu-se inicialmente à filtragem da variável “área do conhecimento”, com seleção exclusiva dos registros classificados como “Enfermagem” e exclusão dos demais. Em seguida, os dados extraídos das diferentes planilhas foram agregados em uma única base para organização e análise. Após a agregação, os registros foram revisados quanto à consistência das variáveis analisadas, não sendo identificados dados faltantes.

### Variáveis do Estudo

As variáveis extraídas foram: ano da titulação; faixa etária do titulado; país de nacionalidade; grau acadêmico (mestrado acadêmico, mestrado profissional, doutorado acadêmico ou doutorado profissional); e instituição de ensino superior (IES) formadora. Na análise de tendência, a variável dependente foi o percentual anual de titulações, calculado como a razão entre o número de titulados em cada ano e o total de titulações do respectivo nível de formação no período analisado, multiplicado por 100. A variável independente correspondeu ao ano da titulação.

### Análise e Tratamento dos Dados

Os dados foram organizados no *Microsoft Excel* ^®^. As estatísticas descritivas foram calculadas no *software* JASP (versão 0.95.4; Amsterdã, Holanda). A distribuição espacial das titulações foi representada por meio de mapas coropléticos elaborados no software QGIS (versão 3.44.4), utilizando *shapefiles* das unidades federativas disponibilizados pelo Instituto Brasileiro de Geografia e Estatística (IBGE). Os dados foram agregados por estado e representados por meio de frequências absolutas e relativas, permitindo a visualização da distribuição espacial das titulações no território brasileiro. Adicionalmente, em cada região foram destacadas as duas IES que mais titularam mestres e doutores na área de enfermagem.

A análise de tendência temporal foi conduzida a partir de séries anuais independentes para as titulações de mestrado e de doutorado no *Joinpoint Regression Program* (versão 5.3.0). O método emprega regressão segmentada com transformação logarítmica dos valores e o teste de permutação de Monte Carlo, com nível de significância de p < 0,05, para identificar pontos de mudança ao longo do período analisado^(8)^. As tendências foram expressas pela Variação Percentual Anual Média (*Average Annual Percentage Change* – AAPC), com intervalo de confiança de 95% (IC95%). A AAPC é uma medida resumida da tendência ao longo de um intervalo temporal predefinido, calculada como a média ponderada das variações percentuais anuais do modelo de pontos de inflexão^(9)^.

A fórmula da AAPC é expressa como: AAPC = {exp (Σwibi/Σwi) − 1} × 100, em que bi representa o coeficiente de regressão de cada intervalo temporal identificado pelo modelo e wi, a largura do intervalo, definida pelo número de anos de cada segmento^(9)^. Valores negativos de AAPC indicam tendência decrescente e valores positivos, tendência crescente, desde que estatisticamente significativos (p < 0,05). Na ausência de significância estatística (p > 0,05), a série é interpretada como estacionária^(10)^.

### Aspectos Éticos

Por tratar-se de uma investigação conduzida com dados secundários de domínio público e acesso irrestrito, dispensou-se a aprovação por Comitê de Ética em Pesquisa, conforme estabelecido na Resolução nº 510/2016 do Conselho Nacional de Saúde^(11)^.

## RESULTADOS

Entre 2004 e 2023, foram identificadas 22.923 titulações de pós-graduação na área de enfermagem no Brasil, das quais 17.174 (74,92%) corresponderam ao mestrado e 5.749 (25,08%) ao doutorado, com maior concentração nas regiões Sudeste (49,74%), Nordeste (22,20%) e Sul (20,78%). Entre os titulados, predominaram as faixas etárias de 30 a 39 anos (n = 10.390; 45,32%) e de 20 a 29 anos (n = 6.789; 29,62%) (Tabela 1).

Em relação ao grau acadêmico, observaram-se maiores proporções de mestrado acadêmico (n = 14.294; 62,36%) e de doutorado acadêmico (n = 5.743; 25,05%). Quanto à natureza jurídica das IES formadoras, a maioria das titulações ocorreu em instituições públicas federais (n = 13.654; 59,56%) (Tabela 1). A Figura 1 apresenta a distribuição temporal das titulações por modalidade no período analisado, evidenciando a variação anual do número de títulos concedidos ao longo da série histórica, com redução observada em 2020.

**Tabela 1:** Distribuição dos titulados segundo região, faixa etária, grau acadêmico e natureza jurídica da instituição – Brasil, 2004–2023.

Variáveis	Região do Brasil					Brasil
	Norte	Nordeste	Centro-Oeste	Sudeste	Sul	
	n = 362 (%)	n = 5.090 (%)	n = 1.304 (%)	n = 11.403 (%)	n = 4.764 (%)	n = 22.923 (%)
*Faixa etária dos titulados*						
19 ou menos	0 (0,00)	0 (0,00)	0 (0,00)	0 (0,00)	1 (0,03)	1 (0,00)
20 a 29 anos	134 (37,02)	1.776 (34,88)	477 (36,58)	2.836 (24,88)	1.468 (30,82)	6.789 (29,62)
30 a 39 anos	156 (43,10)	2.230 (43,79)	572 (43,86)	5.303 (46,51)	2.129 (44,68)	10.390 (45,32)
40 a 49 anos	52 (14,36)	747 (16,67)	211 (16,19)	2.338 (20,50)	860 (18,05)	4.208 (18,36)
50 a 59 anos	17 (4,70)	199 (3,90)	38 (2,91)	793 (6,96)	275 (5,77)	1.322 (5,77)
60 a 69 anos	3 (0,82)	35 (0,68)	6 (0,46)	125 (1,09)	30 (0,62)	199 (0,87)
70 ou mais	0 (0,00)	5 (0,08)	0 (0,00)	8 (0,06)	1 (0,03)	14 (0,06)
*Grau acadêmico*						
Mestrado acadêmico	306 (84,53)	3.377 (66,35)	987 (75,69)	6.852 (60,09)	2.772 (58,19)	14.294 (62,36)
Mestrado profissional	56 (15,47)	676 (13,28)	148 (11,35)	1.134 (9,94)	866 (18,18)	2.880 (12,56)
Doutorado acadêmico	0 (0,00)	1.037 (20,37)	169 (12,96)	3.414 (29,94)	1.123 (23,57)	5.743 (25,05)
Doutorado profissional	0 (0,00)	0 (0,00)	0 (0,00)	3 (0,03)	3 (0,06)	6 (0,03)
*Classificação da natureza jurídica da instituição do programa de pós-graduação*						
Federal	225 (62,15)	3.559 (69,92)	1.016 (77,91)	5.274 (50,27)	3.580 (75,15)	13.654 (59,56)
Estadual	137 (37,85)	1.172 (23,03)	148 (11,35)	5.732 (46,25)	885 (18,58)	8.074 (35,23)
Particular	0 (0,00)	359 (7,05)	140 (10,74)	397 (3,48)	299 (6,28)	1.195 (5,21)

**Figura 1:** Titulados na área de enfermagem, por grau acadêmico (n = 22.923) – Brasil, 2004–2023.

Em relação às IES que mais outorgaram títulos de mestre e doutor, destacaram-se, na região norte, a Universidade Federal do Pará (n = 133) e a Universidade do Estado do Pará (n = 102). No Nordeste, as maiores frequências foram observadas na Universidade Federal do Ceará (n = 1.130) e na Universidade Federal da Paraíba (n = 654). No Centro-Oeste, sobressaíram a Universidade Federal de Goiás (n = 418) e a Universidade Federal do Mato Grosso (n = 226). No Sudeste, a formação concentrou-se na Universidade de São Paulo (n = 4.012) e na Universidade Federal do Rio de Janeiro (n = 903). Na região Sul, destacaram-se a Universidade Federal de Santa Catarina (UFSC) (n = 1.118) e a Universidade Federal do Paraná (n = 571). Quanto à nacionalidade dos titulados, 92,86% (n = 22.717) eram brasileiros e 7,14% (n= 206) estrangeiros, provenientes de 29 países. Entre os estrangeiros, 98 obtiveram o mestrado e 108 o doutorado. As maiores frequências foram observadas entre titulados oriundos do México (n = 44), Chile (n = 28), Colômbia (n = 24) e Peru (n = 23) (Figura 2).

**Figura 2:** Distribuição dos titulados na área de enfermagem no Brasil segundo país de nacionalidade, 2004–2023.

A distribuição espacial das titulações de mestrado evidenciou acentuada concentração nas regiões Sul e Sudeste, especialmente nos estados de São Paulo, Rio de Janeiro, Minas Gerais, Rio Grande do Sul e Paraná, integrantes da classe de maior intensidade. No Nordeste, destacaram-se Bahia, Ceará e Paraíba. Em contraposição, Espírito Santo, Sergipe, Amazonas, Maranhão e grande parte dos estados da região Norte apresentaram quantitativos reduzidos, ao passo que Acre, Amapá, Rondônia, Roraima e Tocantins não registraram titulações de mestrado no período analisado (Figuras 3A e 3B).

**Figura 3:** Distribuição espacial das titulações de mestrado e doutorado na área de enfermagem por unidade federativa – Brasil, 2004–2023.

*A = frequência absoluta (mestrado); ^†^B = frequência relativa (mestrado); ^‡^C = frequência absoluta (doutorado); ^§^D = frequência relativa (doutorado)

No que tange às titulações de doutorado, observa-se, nas Figuras 3C e 3D, concentração nos estados de São Paulo, Rio de Janeiro, Rio Grande do Sul e Santa Catarina. No Nordeste, o Ceará destacou-se como principal polo formador, enquanto Bahia e Paraíba apresentaram níveis intermediários. Em contraste, nenhuma titulação de doutorado em enfermagem por instituições locais foi registrada nos estados da região Norte (Acre, Amapá, Amazonas, Pará, Rondônia, Roraima e Tocantins) nem nos estados do Maranhão, Alagoas, Sergipe, Espírito Santo e Mato Grosso do Sul no período analisado (Figuras 3C e 3D).

Ao longo dos 20 anos analisados, as titulações de mestrado em enfermagem apresentaram tendência temporal crescente e estatisticamente significativa (AAPC: 7,55%; p < 0,000), com crescimento registrado em todas as cinco regiões e destaque para a região Norte (AAPC: 16,40%; p < 0,000). No doutorado, verificou-se tendência nacional igualmente crescente e significativa (AAPC: 8,31% ao ano; p < 0,000), observada em quatro regiões, com maior AAPC no Centro-Oeste (15,96%; p < 0,000), conforme apresentado na Tabela 2.

**Tabela 2:** Variação percentual média anual das titulações de mestrado e doutorado na área de enfermagem no Brasil e regiões – Brasil, 2004–2023.

Variáveis	Período	AAPC*	Tendência	IC 95%^†^	^‡^p-valor
*Mestrado*					
Brasil	2004–2023	7,55	Crescente	6,60; 8,90	< 0,000
Norte	2012–2023	16,40	Crescente	9,82; 26,57	< 0,000
Nordeste	2004–2023	10,78	Crescente	9,54; 12,55	< 0,000
Centro-Oeste	2004–2023	14,23	Crescente	12,70; 17,19	< 0,000
Sudeste	2004–2023	4,48	Crescente	3,56; 5,53	< 0,000
Sul	2004–2023	9,25	Crescente	6,86; 13,67	< 0,000
*Doutorado* ^§^					
Brasil	2004–2023	8,31	Crescente	6,89; 9,72	< 0,000
Nordeste	2004–2023	15,53	Crescente	13,14; 20,49	< 0,000
Centro-Oeste	2013–2023	15,96	Crescente	8,21; 29,73	< 0,000
Sudeste	2004–2023	6,44	Crescente	4,31; 9,16	< 0,000
Sul	2004–2023	14,67	Crescente	12,13; 20,80	< 0,000

*AAPC = Variação Percentual Média Anual;

^†^IC 95% = Intervalo de Confiança de 95%;

^‡^p < 0,05;

^§^Doutorado = a região Norte não foi incluída devido à ausência de titulações nesse nível registradas na área de enfermagem no período analisado.

## DISCUSSÃO

Os dados desvelaram proeminência de titulações de mestrado e doutorado nas regiões Sudeste, Nordeste e Sul, com predominância da modalidade acadêmica e maior participação das IES públicas federais na outorga dos títulos. Esse padrão é coerente com o documento de área da enfermagem, que aponta a centralização de PPGENFs nessas regiões e a prevalência da modalidade acadêmica^(3)^. As IES públicas federais reúnem a maior parcela dos programas stricto sensu no país, o que contribui para explicar sua centralidade na formação de mestres e doutores neste estudo^(12)^.

O protagonismo da região Sudeste na formação stricto sensu em enfermagem pode ser compreendido a partir do seu processo histórico de institucionalização, considerando que os primeiros PPGENFs foram implantados nessa região e, em seguida, expandiram-se para as regiões Sul e Nordeste^(1)^. Sob essa perspectiva, a distribuição das titulações observada nos resultados reflete trajetórias históricas de consolidação e expansão da pós-graduação na área, uma vez que regiões com institucionalização mais precoce tendem a concentrar maior volume de programas e, consequentemente, de titulações. Essa trajetória situa-se em um processo mais amplo de expansão da PGENF nas Américas, cujos primeiros programas foram iniciados nos Estados Unidos (EUA) na década de 1930, expandindo-se posteriormente para países latino-americanos. Nesse sentido, destaca-se a influência norte-americana na consolidação da PGENF brasileira, evidenciada pela especialização de docentes brasileiros nos EUA e pelo apoio financeiro de instituições como a Fundação Kellogg tanto para a formação de recursos humanos quanto para a estruturação dos primeiros programas no país^(2)^.

Na região Norte, o primeiro mestrado em enfermagem teve início apenas em 2010, em associação entre a Universidade do Estado do Pará (UEPA) e a Universidade Federal do Amazonas (UFAM)^(13)^, evidenciando assimetria histórica de várias décadas em relação às regiões Sudeste, Sul e Nordeste, onde cursos de mestrado já estavam em funcionamento desde a década de 1970^(1)^. Ademais, no período analisado, não foram identificadas outorgas de títulos de doutorado em enfermagem por instituições da região, uma vez que o primeiro curso nesse nível foi aprovado apenas em 2023, também vinculado à UEPA/UFAM, com início da primeira turma em 2024. A implantação desse curso constitui um marco na consolidação da formação avançada regional, ao ampliar a qualificação de recursos humanos em nível de doutorado e fortalecer a produção científica e a inovação em enfermagem no contexto amazônico^(14,15)^. A incipiência das titulações de Doutorado Profissional (DP) observada neste estudo pode ser explicada pela recente implementação dessa modalidade na enfermagem brasileira. Os cursos de DP tiveram início em 2019, enquanto as primeiras defesas ocorreram apenas em 2023, na UFSC e na Universidade Estadual Paulista (UNESP)^(16)^.

As assimetrias regionais identificadas não constituem novidade e são reconhecidas pela própria área, que aponta a persistência de desafios relacionados à concentração de programas nas capitais e à baixa oferta de cursos na região Norte, no interior do Nordeste e no Centro-Oeste. Como estratégias para a redução dessas assimetrias, destacam-se a interiorização da pós-graduação, a ampliação da oferta de cursos de doutorado e o fortalecimento de estratégias indutivas, como os Programas Interinstitucionais de Mestrado e Doutorado (chamados respectivamente *Minter* e *Dinter*) e o estabelecimento de parcerias entre PPGENFs com diferentes níveis de maturidade^(3)^.

Neste estudo, observou-se redução no número de titulações em 2020. Esse achado não se configura como um fenômeno isolado, tendo em vista que evidência da pós-graduação brasileira aponta diminuição das titulações em diferentes áreas, associada às dificuldades para a conclusão dos cursos durante a pandemia de COVID-19^(17)^. Dados da enfermagem provenientes da pesquisa *Brasil: mestres e doutores* corroboram esse panorama, indicando redução nas taxas de crescimento das titulações em 2020, tanto no mestrado (–25,54%) quanto no doutorado (–24,9%)^(18)^. À luz desse contexto, o decréscimo observado pode ter sido influenciado por medidas institucionais adotadas pela CAPES, como a flexibilização dos prazos de titulação e a prorrogação excepcional das bolsas no período pandêmico^(19, 20, 21)^. Paralelamente, os impactos psicossociais da pandemia também se mostraram relevantes, associados a efeitos na saúde mental dos pós-graduandos brasileiros, como desmotivação, dificuldades de concentração, insônia e elevados níveis de ansiedade e depressão, além de prejuízos na produção acadêmica e no cumprimento de prazos, fatores que podem ter influenciado a progressão acadêmica^(22, 23, 24)^.

Quanto à nacionalidade dos titulados, os dados indicam que o Brasil tem contribuído exponencialmente para a formação de mestres e doutores estrangeiros, especialmente oriundos da América Latina. O desenvolvimento de cursos interinstitucionais de pós-graduação constitui uma das principais formas pelas quais essa contribuição pode ocorrer^(25)^. Nesse âmbito, a área de enfermagem aponta o compromisso de continuar contribuindo solidariamente com propostas da Organização Pan-Americana da Saúde (OPAS) e da Organização Mundial da Saúde (OMS) voltadas à formação de enfermeiros doutores para a América Latina e o Caribe, incluindo a adoção de mecanismos de atração de estudantes da região e o fortalecimento da integração Sul–Sul, com vistas à nucleação de doutores em seus países de origem e à promoção do desenvolvimento científico e tecnológico na região^(3)^.

No contexto ibero-americano, a formação doutoral em enfermagem tem se caracterizado pela expansão progressiva dos programas, acompanhada da persistência de desigualdades regionais em sua oferta. Nesse cenário, o Brasil desponta como país pioneiro nessa modalidade formativa, com início em 1982, e como principal polo regional, concentrando o maior número de PPGENFs em funcionamento^(26)^. No âmbito específico da América Latina, observa-se crescimento no número de programas, com Brasil, Peru e México respondendo pela maior parte da oferta. Apesar dessa expansão, persistem desafios que comprometem a qualidade e a sustentabilidade da formação doutoral, a saber: a distribuição geográfica desigual dos programas, as limitações de financiamento para pesquisa, a baixa articulação interinstitucional e a proeminência de doutorados orientados majoritariamente à pesquisa acadêmica, com reduzida inserção de modelos voltados à prática avançada^(27)^.

Nessa perspectiva, a nucleação de doutores na América Latina pode representar estratégia relevante para redução de assimetrias na formação avançada em enfermagem, sobretudo em países com oferta ainda limitada de cursos doutorais. A formação desses profissionais em programas brasileiros pode favorecer a expansão da capacidade regional de pesquisa e formação stricto sensu. Contudo, os impactos desse processo dependem de condições estruturais nos países de origem, incluindo políticas de valorização profissional, financiamento científico e suporte institucional para consolidação de novos programas de pós-graduação.

Para além desses aspectos, a literatura aponta a necessidade de intensificar a cooperação internacional e o desenvolvimento de pesquisas multicêntricas como estratégias para o fortalecimento da formação doutoral em enfermagem e para a consolidação da área em diferentes contextos^(28)^. Nessa mesma direção, evidenciam-se desigualdades que extrapolam a dimensão territorial, alcançando aspectos sociais e raciais, com barreiras persistentes para populações minoritárias e lacunas no suporte a estudantes com identidades interseccionais, o que reforça a centralidade da equidade como eixo estruturante da educação doutoral em enfermagem^(29)^.

A formação em nível de mestrado e doutorado em enfermagem deve ser compreendida para além da obtenção do diploma, envolvendo a constituição de sujeitos capazes de exercer liderança científica, política e social, produzir conhecimento relevante e responder aos desafios contemporâneos da profissão e da sociedade. Nesse contexto, a PGENF insere-se em um contexto de profundas transformações científicas, tecnológicas e sociais, demandando mestres e doutores comprometidos com a inovação, a sustentabilidade, a equidade e a produção de impactos concretos no cuidado, na gestão e na formulação de políticas em saúde. Assim, a expansão e a consolidação da formação stricto sensu devem ser acompanhadas de reflexões contínuas sobre o papel desses profissionais na construção da ciência da enfermagem e no fortalecimento de seu compromisso ético, social e político^(30)^.

Este estudo demonstrou aumento das titulações de mestrado e doutorado no país ao longo do período analisado, inclusive nas regiões historicamente menos contempladas pela oferta de programas. Esse avanço, embora relevante, suscita reflexões que extrapolam a dimensão quantitativa da formação avançada. Quantos titulados seriam necessários para promover uma distribuição mais equitativa da formação no território nacional? E, sobretudo, seria a ampliação do número de mestres e doutores suficiente para enfrentar as assimetrias regionais historicamente construídas na enfermagem brasileira?

Essas questões evidenciam contradições, incertezas e desafios inerentes à evolução histórica da profissão de enfermagem no Brasil, à trajetória da formação universitária e aos percursos de consolidação da ciência e tecnologia em enfermagem. Do mesmo modo, revelam tensões em relação ao próprio modelo de expansão da pós-graduação, uma vez que o impacto da titulação não depende exclusivamente do volume de profissionais formados, mas de condições estruturais mais amplas, como políticas de fixação e valorização de mestres e doutores, financiamento sustentável da pesquisa, oportunidades de inserção qualificada no ensino, na prática e na gestão em saúde, bem como da capacidade dos territórios de absorver e valorizar esses profissionais. Nesse sentido, a formação stricto sensu, embora fundamental, não garante automaticamente a redução das desigualdades regionais nem a produção de impactos proporcionais na prática profissional, demandando articulação entre políticas educacionais, científicas, de saúde e de desenvolvimento regional. Assim, o desafio não se restringe a formar mais, mas a criar condições para que mestres e doutores produzam efeitos concretos nesses diferentes campos, com vistas a alcançar elevado domínio científico e tecnológico e a consolidar a enfermagem brasileira como referência no cenário internacional.

Este estudo apresenta algumas limitações. A primeira relaciona-se ao uso de dados secundários, o que pode acarretar restrições quanto à qualidade das informações disponíveis. Além disso, a utilização dessa modalidade de base de dados pode limitar análises mais aprofundadas pela restrição das variáveis disponíveis. Acrescenta-se que a análise de tendência baseada em percentuais de titulação pode restringir a compreensão da magnitude das variações observadas ao longo do tempo. Outra limitação refere-se à condução da análise com dados agregados por unidade federativa e região, o que pode atenuar a identificação de desigualdades em escalas geográficas menores, como municípios. Diante disso, estudos futuros podem empregar distintos delineamentos metodológicos, variáveis adicionais e análises complementares com valores absolutos ou taxas ajustadas, a fim de ampliar a compreensão do fenômeno.

O recorte temporal contemplado pela base de dados restringiu-se ao período de 2004 a 2023, não abrangendo a totalidade da formação stricto sensu em enfermagem no contexto brasileiro. Soma-se a isso a escassez de estudos sobre a evolução temporal das titulações em enfermagem, especialmente em perspectiva internacional, o que limitou comparações mais aprofundadas entre os achados deste estudo e contextos com tradição consolidada na PGENF. Ainda assim, os dados analisados permitiram uma avaliação consistente da evolução das titulações em enfermagem ao longo de duas décadas, oferecendo subsídios relevantes para a compreensão da dinâmica da formação avançada na área.

## CONCLUSÃO

Este estudo analisou a tendência temporal e a distribuição espacial das titulações de mestrado e doutorado na área de enfermagem, demonstrando crescimento ao longo do período em todas as regiões do Brasil. Os achados indicam proeminência das titulações em nível de mestrado, majoritariamente na modalidade acadêmica, e centralidade das IES públicas federais na outorga dos títulos. Do ponto de vista espacial, observou-se concentração da formação nas regiões Sudeste, Nordeste e Sul, bem como a ausência de titulações de mestrado e doutorado por instituições locais em alguns estados ao longo do período analisado, especialmente na região Norte.

Apesar do crescimento observado, persistem marcadas assimetrias regionais na formação stricto sensu em enfermagem. Esse cenário reforça a necessidade de estratégias voltadas à descentralização dos PPGENFs, à ampliação da oferta de cursos em regiões historicamente menos contempladas e à interiorização da formação avançada. Os dados apresentados podem, nesse sentido, subsidiar gestores acadêmicos e formuladores de políticas educacionais na definição de ações direcionadas ao fortalecimento de programas em consolidação e à indução de novas ofertas formativas nos níveis de mestrado e doutorado. Ademais, a ampliação das titulações deve ser acompanhada de políticas de valorização, fixação e inserção qualificada de mestres e doutores nos territórios, de modo a potencializar impactos no ensino, na pesquisa, na prática profissional e na gestão em saúde.

## Data Availability

Todo o conjunto de dados que dá suporte aos resultados deste estudo está disponível mediante solicitação ao autor correspondente.

# Translation: Títulos de maestría y doctorado en el área de enfermería en Brasil: estudio ecológico

**DOI:** 10.1590/1980-220X-REEUSP-2026-0179es

**Authors:** Matheus Moraes Silva, José Luís Guedes dos Santos, Gabriela Marcellino de Melo Lanzoni, Alacoque Lorenzini Erdmann

**Affiliations:** 1 Universidade Federal de Santa Catarina, Programa de Pós-Graduação em Enfermagem, Florianópolis, SC, Brasil.

**Keywords:** Enfermería, Educación de Postgrado, Personal de Enfermería, Enseñanza, Investigación en Enfermería

## Abstract

**Objetivo:**

Analizar la tendencia temporal y la distribución espacial de los títulos de maestría y doctorado en el área de enfermería en Brasil.

**Método:**

Estudio ecológico con datos de la Coordinación de Perfeccionamiento del Personal de Nivel Superior, utilizando estadística descriptiva y regresión *Joinpoint* para el análisis de la tendencia temporal.

**Resultados:**

Entre 2004 y 2023, se registraron 22 923 títulos, de los cuales el 74,92 % correspondieron a maestrías y el 25,08 % a doctorados, con mayor concentración en las regiones del Sudeste (49,74 %), Nordeste (22,20 %) y Sur (20,78 %). Los títulos de maestría mostraron una tendencia al alza en Brasil (Variación porcentual anual promedio [AAPC]: 7,55; p < 0,000), con la mayor variación en la región Norte (AAPC: 16,40; p < 0,000). En cuanto al doctorado, también se observó una tendencia al alza en el país (AAPC: 8,31; p < 0,000), con especial destaque para la región Centro-Oeste (AAPC: 15,96; p < 0,000), mientras que la región Norte no registró títulos de este nivel en el período analizado.

**Conclusión:**

Los resultados evidenciaron una tendencia al alza en los títulos de posgrado en el área de enfermería, junto con desigualdades regionales, lo que refuerza la necesidad de estrategias orientadas a la ampliación y la descentralización de la educación de posgrado para reducir las asimetrías en la formación avanzada.

## INTRODUCCIÓN

La posgraduación en enfermería (PGENF) en Brasil se instituyó con el objetivo de capacitar a profesionales para la docencia en la educación superior, impulsar la producción científica y fortalecer las prácticas basadas en la evidencia^(1)^. Este proceso se consolidó con la implementación del primer máster en la Escuela de Enfermería Anna Nery (EEAN), en 1972, y del doctorado en la Escuela de Enfermería de la Universidad de São Paulo (EEUSP), en 1981^(1,2)^.

En las últimas décadas, la posgraduación en esta área ha crecido exponencialmente. Los datos de la Coordinación de Perfeccionamiento del Personal de Nivel Superior (CAPES) indican que, hasta 2024, el país contaba con 86 programas, que sumaban un total de 135 cursos, de los cuales 58 programas y 99 cursos correspondían a la modalidad académica y 28 programas y 36 cursos a la modalidad profesional. La modalidad académica prioriza la producción de conocimiento científico, la formación para la investigación y la capacitación para la docencia, mientras que la profesional hace hincapié en la aplicación del conocimiento en la práctica y el desarrollo de tecnologías e innovaciones en los servicios de salud^(3)^.

Los programas de posgrado en enfermería (PPGENF) son los principales responsables de la generación de conocimiento especializado en el área y contribuyen sustancialmente al avance de la ciencia de la enfermería^(4)^, una posición que se refleja en el primer lugar que ocupa Brasil en producción científica en enfermería en América Latina y entre los diez países con mayor producción mundial, según el *ranking* SCImago de 2024^(5)^.

A la luz de este panorama, resulta relevante comprender cómo se han configurado los títulos de maestría y doctorado en enfermería desde las perspectivas temporal y espacial en el territorio brasileño. Aunque la expansión de los PPGENF brasileños es ampliamente reconocida, aún son escasos los estudios que adoptan los títulos otorgados como objeto central de análisis, especialmente considerando su distribución regional y su evolución a lo largo del tiempo. En este contexto, investigar esta situación puede contribuir a identificar asimetrías en la formación avanzada en enfermería, lo que respalda reflexiones relacionadas con la planificación de los programas de posgrado en enfermería (PPGENF), la formación de recursos humanos y las políticas científicas y educativas del área.

Ante lo expuesto, este estudio tiene como objetivo analizar la tendencia temporal y la distribución espacial de los títulos de maestría y doctorado en el área de enfermería en Brasil.

## MÉTODO

### Diseño y Lugar del Estudio

Se trata de un estudio ecológico desarrollado a partir de datos secundarios extraídos de la CAPES. En este tipo de diseño, las inferencias se limitan al nivel poblacional^(6)^. Las unidades de análisis correspondieron a los 27 estados y a las cinco regiones geográficas de Brasil. El estudio se reportó de acuerdo con las recomendaciones de STROBE (*Strengthening the Reporting of Observational Studies in Epidemiology*).

### Muestra, Criterios de Selección y Fuente de Datos

El estudio se compuso de todos los registros de títulos de maestría y doctorado en enfermería disponibles en el Panel de Datos Abiertos de la CAPES^(7)^. La recolección de datos se llevó a cabo el 15 de noviembre de 2025, mediante la selección del tema “Evaluación de la posgrado *stricto sensu*” y del grupo “Estudiantes de posgrado *stricto sensu* en Brasil”. Se extrajeron archivos en formato CSV (*Comma-Separated Values*) correspondientes al período de 2004 a 2023, que abarca todo el intervalo temporal disponible en la base de datos al momento de la Recolección. Para el procesamiento de los datos, se procedió inicialmente a filtrar la variable “área de conocimiento”, seleccionando exclusivamente los registros clasificados como “Enfermería” y excluyendo los demás. A continuación, los datos extraídos de las diferentes hojas de cálculo se agregaron en una única base para su organización y análisis. Tras la agregación, se revisaron los registros para verificar la consistencia de las variables analizadas, sin que se identificaran datos faltantes.

### Variables sel Estudio

Las variables extraídas fueron: año de obtención del título; rango de edad del titulado; país de nacionalidad; grado académico (maestría académica, maestría profesional, doctorado académico o doctorado profesional); e institución de educación superior (IES) de formación. En el análisis de tendencias, la variable dependiente fue el porcentaje anual de títulos, calculado como la relación entre el número de titulados en cada año y el total de títulos del respectivo nivel de formación en el período analizado, multiplicado por 100. La variable independiente correspondió al año de obtención del título.

### Análisis y Tratamiento de Los Datos

Los datos se organizaron en *Microsoft Excel* ^®^. Las estadísticas descriptivas se calcularon con el *software* JASP (versión 0.95.4; Ámsterdam, Países Bajos). La distribución espacial de los títulos se representó mediante mapas coropléticos elaborados con el software QGIS (versión 3.44.4), utilizando *shapefiles* de las unidades federativas proporcionados por el Instituto Brasileño de Geografía y Estadística (IBGE). Los datos se agregaron por estado y se representaron mediante frecuencias absolutas y relativas, lo que permitió visualizar la distribución espacial de los títulos en el territorio brasileño. Además, en cada región se destacaron las dos instituciones de educación superior que otorgaron más títulos de maestría y doctorado en el área de enfermería.

El análisis de tendencias temporales se llevó a cabo a partir de series anuales independientes para los títulos de maestría y doctorado en el *Joinpoint Regression Program* (versión 5.3.0). El método emplea regresión segmentada con transformación logarítmica de los valores y la prueba de permutación de Monte Carlo, con un nivel de significación de p < 0,05, para identificar puntos de cambio a lo largo del período analizado^(8)^. Las tendencias se expresaron mediante la variación porcentual anual promedio (*Average Annual Percentage Change* – AAPC), con un intervalo de confianza del 95 % (IC95 %). La AAPC es una medida resumida de la tendencia a lo largo de un intervalo temporal predefinido, calculada como el promedio ponderado de las variaciones porcentuales anuales del modelo de puntos de inflexión^(9)^.

La fórmula de la AAPC se expresa como: AAPC = {exp (Σwibi/Σwi) − 1} × 100, donde bi representa el coeficiente de regresión de cada intervalo temporal identificado por el modelo y wi, la amplitud del intervalo, definida por el número de años de cada segmento^(9)^. Los valores negativos de la AAPC indican una tendencia decreciente y los valores positivos, una tendencia creciente, siempre que sean estadísticamente significativos (p < 0,05). En ausencia de significación estadística (p > 0,05), la serie se interpreta como estacionaria^(10)^.

### Aspectos Éticos

Al tratarse de una investigación realizada con datos secundarios de dominio público y acceso irrestrito, no se requirió la aprobación de un Comité de Ética en Investigación, conforme a lo establecido en la Resolución n.º 510/2016 del Consejo Nacional de Salud^(11)^.

## RESULTADOS

Entre 2004 y 2023, se identificaron 22 923 títulos de posgrado en el área de enfermería en Brasil, de los cuales 17 174 (74,92 %) correspondieron a maestrías y 5 749 (25,08 %) al doctorado, con mayor concentración en las regiones Sudeste (49,74 %), Nordeste (22,20 %) y Sur (20,78 %). Entre los titulados, predominaron los grupos de edad de 30 a 39 años (n = 10 390; 45,32 %) y de 20 a 29 años (n = 6 789; 29,62 %) (Tabla 1). En cuanto al nivel académico, se observaron mayores proporciones de maestrías académicas (n = 14 294; 62,36 %) y de doctorados académicos (n = 5 743; 25,05 %). En cuanto a la naturaleza jurídica de las instituciones de educación superior (IES) que otorgaron los títulos, la mayoría de los títulos se otorgaron en instituciones públicas federales (n = 13 654; 59,56 %) (Tabla 1). La Figura 1 presenta la distribución temporal de los títulos por modalidad en el período analizado, lo que pone de manifiesto la variación anual en el número de títulos otorgados a lo largo de la serie histórica, con una reducción observada en 2020.

**Tabla 1:** Distribución de los graduados según región, grupo etario, grado académico y naturaleza jurídica de la institución – Brasil, 2004–2023.

Variables	Región do Brasil					Brasil
	Norte	Nordeste	Centro-Oeste	Sudeste	Sur	
	n = 362 (%)	n = 5.090 (%)	n = 1.304 (%)	n = 11.403 (%)	n = 4.764 (%)	n = 22.923 (%)
*Grupo etario de los graduados*						
19 o menos	0 (0,00)	0 (0,00)	0 (0,00)	0 (0,00)	1 (0,03)	1 (0,00)
20 a 29 años	134 (37,02)	1.776 (34,88)	477 (36,58)	2.836 (24,88)	1.468 (30,82)	6.789 (29,62)
30 a 39 años	156 (43,10)	2.230 (43,79)	572 (43,86)	5.303 (46,51)	2.129 (44,68)	10.390 (45,32)
40 a 49 años	52 (14,36)	747 (16,67)	211 (16,19)	2.338 (20,50)	860 (18,05)	4.208 (18,36)
50 a 59 años	17 (4,70)	199 (3,90)	38 (2,91)	793 (6,96)	275 (5,77)	1.322 (5,77)
60 a 69 años	3 (0,82)	35 (0,68)	6 (0,46)	125 (1,09)	30 (0,62)	199 (0,87)
70 o más	0 (0,00)	5 (0,08)	0 (0,00)	8 (0,06)	1 (0,03)	14 (0,06)
*Grado académico*						
Maestría académica	306 (84,53)	3.377 (66,35)	987 (75,69)	6.852 (60,09)	2.772 (58,19)	14.294 (62,36)
Maestría profesional	56 (15,47)	676 (13,28)	148 (11,35)	1.134 (9,94)	866 (18,18)	2.880 (12,56)
Doctorado académico	0 (0,00)	1.037 (20,37)	169 (12,96)	3.414 (29,94)	1.123 (23,57)	5.743 (25,05)
Doctorado profesional	0 (0,00)	0 (0,00)	0 (0,00)	3 (0,03)	3 (0,06)	6 (0,03)
*Clasificación de la naturaleza jurídica de la institución del programa de posgraduación*						
Federal	225 (62,15)	3.559 (69,92)	1.016 (77,91)	5.274 (50,27)	3.580 (75,15)	13.654 (59,56)
Estadual	137 (37,85)	1.172 (23,03)	148 (11,35)	5.732 (46,25)	885 (18,58)	8.074 (35,23)
Particular	0 (0,00)	359 (7,05)	140 (10,74)	397 (3,48)	299 (6,28)	1.195 (5,21)

**Figura 1:** Graduados en el área de enfermería, por graduación académica (n = 22.923) – Brasil, 2004–2023.

En cuanto a las instituciones de educación superior que más títulos de maestría y doctorado otorgaron, en la región norte se destacaron la Universidad Federal de Pará (n = 133) y la Universidad Estatal de Pará (n = 102). En el noreste, las cifras más altas se observaron en la Universidad Federal de Ceará (n = 1 130) y en la Universidad Federal de Paraíba (n = 654). En el centro-oeste, se destacaron la Universidad Federal de Goiás (n = 418) y la Universidad Federal de Mato Grosso (n = 226). En el Sudeste, la formación se concentró en la Universidad de São Paulo (n = 4.012) y en la Universidad Federal de Río de Janeiro (n = 903). En la región Sur, se destacaron la Universidad Federal de Santa Catarina (UFSC) (n = 1.118) y la Universidad Federal de Paraná (n = 571). En cuanto a la nacionalidad de los titulados, el 92,86 % (n = 22 717) eran brasileños y el 7,14 % (n = 206) extranjeros, procedentes de 29 países. Entre los extranjeros, 98 obtuvieron la maestría y 108 el doctorado. Las mayores frecuencias se observaron entre los titulados procedentes de México (n = 44), Chile (n = 28), Colombia (n = 24) y Perú (n = 23) (Figura 2).

**Figura 2:** Distribución de los graduados en el área de enfermería en Brasil según su nacionalidad, 2004–2023.

La distribución espacial de los títulos de maestría mostró una marcada concentración en las regiones Sur y Sudeste, especialmente en los estados de São Paulo, Río de Janeiro, Minas Gerais, Río Grande del Sur y Paraná, que forman parte de la clase de mayor intensidad. En el Nordeste, se destacaron Bahía, Ceará y Paraíba. Por el contrario, Espírito Santo, Sergipe, Amazonas, Maranhão y gran parte de los estados de la región Norte presentaron cifras reducidas, mientras que Acre, Amapá, Rondônia, Roraima y Tocantins no registraron títulos de maestría en el período analizado (Figuras 3A y 3B).

**Figura 3:** Distribución espacial de los títulos de maestría y doctorado en el área de enfermería por unidad federativa – Brasil, 2004–2023.

*A = frecuencia absoluta (maestría); ^†^B = frecuencia relativa (maestría); ^‡^C = frecuencia absoluta (doctorado); ^§^D = frecuencia relativa (doctorado)

En cuanto a los títulos de doctorado, en las Figuras 3C y 3D se observa una concentración en los estados de São Paulo, Río de Janeiro, Río Grande del Sur y Santa Catarina. En el Nordeste, Ceará se destacó como el principal centro de formación, mientras que Bahía y Paraíba presentaron niveles intermedios. Por el contrario, no se registró ningún título de doctorado en enfermería otorgado por instituciones locales en los estados de la región Norte (Acre, Amapá, Amazonas, Pará, Rondônia, Roraima y Tocantins), ni en los estados de Maranhão, Alagoas, Sergipe, Espírito Santo y Mato Grosso do Sul durante el período analizado (Figuras 3C y 3D).

A lo largo de los 20 años analizados, los títulos de maestría en enfermería mostraron una tendencia temporal al alza y estadísticamente significativa (AAPC: 7,55 %; p < 0,000), con un crecimiento registrado en las cinco regiones y un destaque especial en la región Norte (AAPC: 16,40 %; p < 0,000). En el caso de los doctorados, se observó una tendencia nacional igualmente creciente y significativa (AAPC: 8,31 % anual; p < 0,000), presente en cuatro regiones, con la mayor AAPC en la región Centro-Oeste (15,96 %; p < 0,000), tal como se presenta en la Tabla 2.

**Tabla 2:** Variación porcentual media anual de las graduaciones de maestría y doctorado en el área de enfermería, Brasil y regiones – Brasil, 2004–2023.

Variables	Período	AAPC*	Tendencia	IC 95%^†^	^‡^p–valor
*Maestría*					
Brasil	2004–2023	7,55	Creciente	6,60; 8,90	< 0,000
Norte	2012–2023	16,40	Creciente	9,82; 26,57	< 0,000
Nordeste	2004–2023	10,78	Creciente	9,54; 12,55	< 0,000
Centro–Oeste	2004–2023	14,23	Creciente	12,70; 17,19	< 0,000
Sudeste	2004–2023	4,48	Creciente	3,56; 5,53	< 0,000
Sur	2004–2023	9,25	Creciente	6,86; 13,67	< 0,000
*Doctorado* ^§^					
Brasil	2004–2023	8,31	Creciente	6,89; 9,72	< 0,000
Nordeste	2004–2023	15,53	Creciente	13,14; 20,49	< 0,000
Centro–Oeste	2013–2023	15,96	Creciente	8,21; 29,73	< 0,000
Sudeste	2004–2023	6,44	Creciente	4,31; 9,16	< 0,000
Sur	2004–2023	14,67	Creciente	12,13; 20,80	< 0,000

*AAPC = Variación Porcentual Media Anual;

^†^IC 95% = Intervalo de Confianza de 95%;

^‡^p < 0,05;

^§^Doctorado = la región Norte no fue incluida por la ausencia de graduaciones de ese nivel registradas en el área de enfermería en el período analizado.

## DISCUSIÓN

Los datos revelaron un mayor número de títulos de maestría y doctorado en las regiones del Sudeste, Nordeste y Sur, con predominio de la modalidad académica y una mayor participación de las instituciones de educación superior públicas federales en la otorgación de los títulos. Este patrón concuerda con el documento de referencia del área de enfermería, que señala la centralización de los programas de posgrado en enfermería (PPGENF) en esas regiones y el predominio de la modalidad académica^(3)^. Las instituciones de educación superior públicas federales concentran la mayor parte de los programas de posgrado de tipo “stricto sensu” en el país, lo que ayuda a explicar su papel central en la formación de maestros y doctores en este estudio^(12)^.

El protagonismo de la región Sudeste en la formación de posgrado en enfermería puede entenderse a partir de su proceso histórico de institucionalización, considerando que los primeros programas de posgrado en enfermería (PPGENF) se implementaron en esa región y, posteriormente, se expandieron hacia las regiones Sur y Nordeste^(1)^. Desde esta perspectiva, la distribución de los títulos observada en los resultados refleja las trayectorias históricas de consolidación y expansión de la posgrado en el área, ya que las regiones con una institucionalización más temprana tienden a concentrar un mayor volumen de programas y, en consecuencia, de títulos. Esta trayectoria se inscribe en un proceso más amplio de expansión de la PGENF en las Américas, cuyos primeros programas se iniciaron en los Estados Unidos (EE. UU.) en la década de 1930, expandiéndose posteriormente a países latinoamericanos. En este sentido, se destaca la influencia estadounidense en la consolidación de la PGENF brasileña, evidenciada por la especialización de docentes brasileños en los EE. UU. y por el apoyo financiero de instituciones como la Fundación Kellogg, tanto para la formación de recursos humanos como para la estructuración de los primeros programas en el país^(2)^.

En la región Norte, la primera maestría en enfermería se inició recién en 2010, mediante una asociación entre la Universidad Estatal de Pará (UEPA) y la Universidad Federal de Amazonas (UFAM)^(13)^, lo que pone de manifiesto una asimetría histórica de varias décadas en relación con las regiones Sudeste, Sur y Nordeste, donde los programas de maestría ya estaban en funcionamiento desde la década de 1970^(1)^. Además, en el período analizado, no se identificó la concesión de títulos de doctorado en enfermería por parte de instituciones de la región, ya que el primer programa de este nivel se aprobó recién en 2023, también vinculado a la UEPA/UFAM, y la primera promoción comenzó en 2024. La implementación de este programa constituye un hito en la consolidación de la formación avanzada regional, al ampliar la capacitación de recursos humanos a nivel de doctorado y fortalecer la producción científica y la innovación en enfermería en el contexto amazónico^(14,15)^. La etapa inicial de los títulos de Doctorado Profesional (DP) en Enfermería observada en este estudio puede explicarse por la reciente implementación de esta modalidad en la enfermería brasileña. Los programas de DP comenzaron en 2019, mientras que las primeras defensas de tesis se realizaron recién en 2023, en la UFSC y en la Universidad Estatal Paulista (UNESP)^(16)^.

Las asimetrías regionales identificadas no son una novedad y son reconocidas por el propio sector, que señala la persistencia de desafíos relacionados con la concentración de programas en las capitales y la escasa oferta de cursos en la región Norte, en el interior del Nordeste y en el Centro-Oeste. Como estrategias para reducir estas asimetrías, se destacan la expansión de la posgrado hacia el interior del país, la ampliación de la oferta de cursos de doctorado y el fortalecimiento de estrategias inductivas, como los Programas Interinstitucionales de Maestría y Doctorado (denominados respectivamente *Minter* y *Dinter*) y el establecimiento de alianzas entre PPGENFs con diferentes niveles de madurez^(3)^.

En este estudio, se observó una reducción en el número de títulos otorgados en 2020. Este hallazgo no constituye un fenómeno aislado, ya que la evidencia sobre la posgrado brasileña apunta a una disminución de los títulos en diferentes áreas, asociada a las dificultades para concluir los cursos durante la pandemia de COVID-19^(17)^. Los datos sobre enfermería procedentes de la investigación *Brasil: maestros y doctores* corroboran este panorama, indicando una reducción en las tasas de crecimiento de las titulaciones en 2020, tanto en la maestría (–25,54 %) como en el doctorado (–24,9 %)^(18)^. A la luz de este contexto, la disminución observada pudo haber sido influenciada por medidas institucionales adoptadas por la CAPES, como la flexibilización de los plazos de titulación y la prórroga excepcional de las becas durante el período de la pandemia^(19, 20, 21)^. Paralelamente, los impactos psicosociales de la pandemia también resultaron relevantes, asociados a efectos en la salud mental de los estudiantes de posgrado brasileños, como la desmotivación, las dificultades de concentración, el insomnio y los altos niveles de ansiedad y depresión, además de perjuicios en la producción académica y en el cumplimiento de los plazos, factores que pueden haber influido en la progresión académica^(22, 23, 24)^.

En cuanto a la nacionalidad de los titulados, los datos indican que Brasil ha contribuido de manera exponencial a la formación de maestros y doctores extranjeros, especialmente procedentes de América Latina. El desarrollo de cursos interinstitucionales de posgrado constituye una de las principales formas en que puede darse esta contribución^(25)^. En este ámbito, el área de enfermería se compromete a seguir contribuyendo solidariamente con las propuestas de la Organización Panamericana de la Salud (OPS) y de la Organización Mundial de la Salud (OMS) orientadas a la formación de enfermeros con doctorado para América Latina y el Caribe, lo que incluye la adopción de mecanismos para atraer a estudiantes de la región y el fortalecimiento de la integración Sur -Sur, con miras a la concentración de doctores en sus países de origen y a la promoción del desarrollo científico y tecnológico en la región^(3)^.

En el contexto iberoamericano, la formación doctoral en enfermería se ha caracterizado por la expansión progresiva de los programas, acompañada de la persistencia de desigualdades regionales en su oferta. En este escenario, Brasil se destaca como país pionero en esta modalidad formativa, que comenzó en 1982, y como principal polo regional, al concentrar el mayor número de programas de posgrado en enfermería (PPGENF) en funcionamiento^(26)^. En el ámbito específico de América Latina, se observa un crecimiento en el número de programas, siendo Brasil, Perú y México los que representan la mayor parte de la oferta.

A pesar de esta expansión, persisten desafíos que comprometen la calidad y la sostenibilidad de la formación doctoral, a saber: la distribución geográfica desigual de los programas, las limitaciones de financiamiento para la investigación, la escasa articulación interinstitucional y el predominio de doctorados orientados mayoritariamente a la investigación académica, con una inserción reducida de modelos enfocados en la práctica avanzada^(27)^.

Desde esta perspectiva, la concentración de doctores en América Latina puede representar una estrategia relevante para reducir las asimetrías en la formación avanzada en enfermería, sobre todo en países con una oferta aún limitada de programas de doctorado. La formación de estos profesionales en programas brasileños puede favorecer la expansión de la capacidad regional de investigación y formación stricto sensu. Sin embargo, los impactos de este proceso dependen de las condiciones estructurales en los países de origen, incluyendo políticas de valorización profesional, financiamiento científico y apoyo institucional para la consolidación de nuevos programas de posgrado.

Más allá de estos aspectos, la literatura señala la necesidad de intensificar la cooperación internacional y el desarrollo de investigaciones multicéntricas como estrategias para fortalecer la formación doctoral en enfermería y para consolidar el área en diferentes contextos^(28)^. En esa misma línea, se evidencian desigualdades que trascienden la dimensión territorial y abarcan aspectos sociales y raciales, con barreras persistentes para las poblaciones minoritarias y brechas en el apoyo a los estudiantes con identidades interseccionales, lo que refuerza la importancia central de la equidad como eje estructurante de la educación de doctorado en enfermería^(29)^.

La formación a nivel de maestría y doctorado en enfermería debe entenderse más allá de la obtención del título, implicando la formación de sujetos capaces de ejercer liderazgo científico, político y social, producir conocimiento relevante y responder a los desafíos contemporáneos de la profesión y de la sociedad. En este contexto, la PGENF se inscribe en un panorama de profundas transformaciones científicas, tecnológicas y sociales, que exige que los maestros y doctores estén comprometidos con la innovación, la sostenibilidad, la equidad y la generación de impactos concretos en la atención, la gestión y la formulación de políticas en salud. Por lo tanto, la expansión y la consolidación de la formación de posgrado deben ir acompañadas de reflexiones continuas sobre el papel de estos profesionales en la construcción de la ciencia de la enfermería y en el fortalecimiento de su compromiso ético, social y político^(30)^.

Este estudio demostró un aumento de los títulos de maestría y doctorado en el país a lo largo del período analizado, incluso en las regiones históricamente menos contempladas por la oferta de programas. Este avance, aunque relevante, suscita reflexiones que van más allá de la dimensión cuantitativa de la formación de posgrado. ¿Cuántos titulados se necesitarían para promover una distribución más equitativa de la formación en el territorio nacional? Y, sobre todo, ¿sería suficiente la ampliación del número de maestros y doctores para hacer frente a las asimetrías regionales históricamente construidas en la enfermería brasileña?

Estas cuestiones ponen de manifiesto contradicciones, incertidumbres y desafíos inherentes a la evolución histórica de la profesión de enfermería en Brasil, a la trayectoria de la formación universitaria y a los procesos de consolidación de la ciencia y la tecnología en enfermería. Del mismo modo, revelan tensiones en relación con el propio modelo de expansión de la posgrado, ya que el impacto de la titulación no depende exclusivamente del volumen de profesionales formados, sino de condiciones estructurales más amplias, como las políticas de retención y valorización de maestros y doctores, el financiamiento sostenible de la investigación, las oportunidades de inserción calificada en la enseñanza, la práctica y la gestión en salud, así como de la capacidad de los territorios para absorber y valorizar a estos profesionales.

En este sentido, la formación de posgrado stricto sensu, aunque fundamental, no garantiza automáticamente la reducción de las desigualdades regionales ni la generación de impactos proporcionales en la práctica profesional, lo que exige una articulación entre las políticas educativas, científicas, de salud y de desarrollo regional. Por lo tanto, el desafío no se limita a formar a más personas, sino a crear las condiciones para que los maestros y doctores generen efectos concretos en estos diferentes campos, con el fin de alcanzar un alto dominio científico y tecnológico y consolidar la enfermería brasileña como referencia en el escenario internacional.

Este estudio presenta algunas limitaciones. La primera se relaciona con el uso de datos secundarios, lo que puede acarrear restricciones en cuanto a la calidad de la información disponible. Además, el uso de este tipo de base de datos puede limitar los análisis más profundos debido a la restricción de las variables disponibles. Cabe agregar que el análisis de tendencias basado en porcentajes de titulación puede limitar la comprensión de la magnitud de las variaciones observadas a lo largo del tiempo. Otra limitación se refiere a la realización del análisis con datos agregados por unidad federativa y región, lo que puede dificultar la identificación de desigualdades en escalas geográficas más pequeñas, como los municipios. Ante esto, los estudios futuros podrían emplear distintos diseños metodológicos, variables adicionales y análisis complementarios con valores absolutos o tasas ajustadas, con el fin de ampliar la comprensión del fenómeno.

El período de tiempo contemplado por la base de datos se limitó al periodo de 2004 a 2023, sin abarcar la totalidad de la formación de posgrado en enfermería en el contexto brasileño. A esto se suma la escasez de estudios sobre la evolución temporal de los títulos de enfermería, especialmente desde una perspectiva internacional, lo que limitó comparaciones más profundas entre los hallazgos de este estudio y contextos con una tradición consolidada en la PGENF. Aun así, los datos analizados permitieron una evaluación consistente de la evolución de los títulos de posgrado en enfermería a lo largo de dos décadas, ofreciendo información relevante para comprender la dinámica de la formación avanzada en el área.

## CONCLUSIÓN

Este estudio analizó la tendencia temporal y la distribución espacial de los títulos de maestría y doctorado en el área de enfermería, demostrando un crecimiento a lo largo del período en todas las regiones de Brasil. Los hallazgos indican un predominio de los títulos de maestría, en su mayoría en la modalidad académica, y un papel central de las instituciones de educación superior (IES) públicas federales en la otorgación de los títulos. Desde el punto de vista espacial, se observó una concentración de la formación en las regiones Sudeste, Noreste y Sur, así como la ausencia de títulos de maestría y doctorado otorgados por instituciones locales en algunos estados a lo largo del período analizado, especialmente en la región Norte.

A pesar del crecimiento observado, persisten marcadas asimetrías regionales en la formación stricto sensu en enfermería. Este panorama refuerza la necesidad de estrategias orientadas a la descentralización de los PPGENF, a la ampliación de la oferta de cursos en regiones históricamente menos atendidas y a la interiorización de la formación avanzada. Los datos presentados pueden, en este sentido, servir de base a los gestores académicos y a los responsables de la formulación de políticas educativas para definir acciones orientadas al fortalecimiento de los programas en proceso de consolidación y a la creación de nuevas ofertas formativas en los niveles de maestría y doctorado. Además, la ampliación de los títulos debe ir acompañada de políticas de valorización, retención e inserción calificada de maestros y doctores en los territorios, a fin de potenciar los impactos en la enseñanza, la investigación, la práctica profesional y la gestión en salud.

## Data Availability

Todo el conjunto de datos que respalda los resultados de este estudio está disponible previa solicitud al autor correspondiente.
